# Efficacy, Feasibility, and Acceptability of an Emotional Competence Tele-Intervention for Mandarin-Speaking Children Aged 5 to 7 Years With Developmental Language Disorder: Pilot Study With an Interrupted Time-Series Design

**DOI:** 10.2196/60333

**Published:** 2025-02-11

**Authors:** Hsin-Hui Lu, Shih-Yuan Liang, Yi-Chia Huang

**Affiliations:** 1 Division of Clinical Psychology, Graduate Institute of Behavioral Sciences, College of Medicine, Chang Gung University Taoyuan City Taiwan; 2 Department of Child Psychiatry, Chang Gung Memorial Hospital at Linkou Taoyuan City Taiwan; 3 Department of Psychology, National Chung Cheng University Chaiyi Taiwan; 4 Department of Child and Adolescent Psychiatry, Chang Gung Memorial Hospital, Kaohsiung Medical Center Kaohsiung Taiwan

**Keywords:** language disorder, pediatrics, evidence-based intervention, telemedicine, tele-practice, visual support, mobile phone

## Abstract

**Background:**

Children with developmental language disorder (DLD) often experience language difficulties that hinder their ability to acquire emotional competence. Poor emotional competence is associated with emotional and behavioral problems in young children.

**Objective:**

This research involved two studies focusing on (1) the emotional competence of Mandarin-speaking children aged 5 to 7 years with DLD and (2) the efficacy, feasibility, and acceptability of a tele-intervention designed to enhance their emotional competence in Taiwan.

**Methods:**

Five children with DLD from study 1 declined to participate in study 2, the emotional competence tele-intervention, and were excluded from the analysis. We compared the emotional competence of 20 Mandarin-speaking children with DLD to that of 24 children with typical language development (TLD). The children with DLD were, on average, aged 5.79 (SD 0.47) years, whereas the children with TLD were, on average, aged 5.93 (SD 0.31) years. We assessed the children’s emotional competence, nonverbal ability, verbal comprehension, vocabulary acquisition, and expressive language skills. In study 2, all children with DLD included in study 1 engaged in an emotional competence tele-intervention. An interrupted time-series design was used to examine their emotional competence. In total, 20 children with DLD provided data on emotional competence evaluated using the Emotional Lexicon Test. These data were individually collected at 3 time points after study 1 (time 1). These phases included baseline (time 1 to time 2), during the tele-intervention (time 2 to time 3), and follow-up (time 3 to time 4), spanning approximately 18 to 20 weeks from time 1 to time 4. Recruitment, retention, and attendance rates were calculated to evaluate the intervention’s feasibility, and participant mood was evaluated after each session to calculate the intervention’s acceptability.

**Results:**

No significant changes in the children’s ability to understand basic or complex emotional terms were observed during the baseline period. However, changes were observed during the tele-intervention period, and these changes remained throughout the follow-up period. With a recruitment rate of 80% (20/25), all participants completed 4 intervention sessions, with retention and attendance rates exceeding 95% (19/20). A total of 90% (18/20) of the participants deemed each session to be acceptable.

**Conclusions:**

Mandarin-speaking children aged 5 to 7 years with DLD exhibited lower emotional competence compared with their counterparts with TLD. Tele-interventions are effective in enhancing the emotional competence of children with DLD, demonstrating feasibility and acceptability for these children and their parents in Taiwan.

## Introduction

### Background

According to the *Diagnostic and Statistical Manual of Mental Disorders, Fifth Edition* (*DSM-5*) [1], language disorder (ie, developmental language disorder [DLD]) is a communication disorder that broadly refers to childhood language difficulties and is not associated with any known biomedical condition [2,3]. These language difficulties can hinder children’s acquisition of emotional competence, including their understanding of the nature and causes of emotions, their own feelings, their physiological reactions to these feelings, and their cognition surrounding an emotion or emotive event [4-7]. Language is strongly associated with emotional competence, as evidenced in our use of prosody to interpret emotional cues, recognize emotions, and understand what others may feel in various situations [8]. When children experience communication difficulties, their opportunities for social learning diminish, making conversations harder to process, misunderstandings more likely, and participation in discussions or play activities more challenging, which can, in turn, hinder their emotional competence [9]. Language also provides access to mental terms such as *think* and *happy*, which help children mentalize and help them identify, understand, express, and regulate their emotions [10]. These capabilities facilitate social interactions and indirectly aid in understanding the mental states of others [11]. Therefore, children with language acquisition difficulties tend to struggle with using these mental terms to learn the emotional or cognitive aspects of theory of mind. Notably, verbal interactions with caregivers play a key role in the development of emotion regulation skills, primarily because discussing emotions helps children connect their emotions to events and learn how to manage these emotions [12]. As children with DLD grow, they struggle to develop the capacity to internally regulate their emotions, with language playing a crucial role in self-reflection, response inhibition, and guiding behavior [13]. Furthermore, in young children with typical language development (TLD), poor emotional competence has been shown to be associated with emotional and behavioral problems [14].

Previous research examining the emotional competence of children with DLD and children with TLD has rarely focused on children who speak Mandarin as their native language [4,11,15]. If studies indicate that the emotional competence of Mandarin-speaking children with DLD lags behind that of their peers with TLD, as is the case for non–Mandarin-speaking children with DLD, this finding will support the presence of cross-linguistic and cross-cultural consistency in the emotional challenges faced by children with DLD. In children with developmental delays, speech and language delays are the most common disabilities [16]. DLD is a highly prevalent neurodevelopmental disability in Mandarin-speaking children [17,18], highlighting the importance of focusing on the emotional competence of this population. Therefore, further research is needed to evaluate the emotional competence of Mandarin-speaking children with DLD and determine whether early intervention is necessary.

Tele-interventions constitute a promising tool that offers a timely, accessible, and cost-effective solution for overcoming barriers to medical service delivery, such as transportation issues, shortage of skilled therapists, and insufficient facilities in rural areas [19]. This approach is particularly significant given that the special needs of children with DLD living in rural areas are often less adequately addressed than those of children living in urban areas [20]. Adopting tele-practice for early interventions holds great potential to address these disparities and support the development of emotional competence in children with DLD in underserved rural areas [19]. In Taiwan, the procedures, advantages, and challenges associated with implementing such interventions remain unclear. Therefore, in this study, we examined the efficacy, feasibility, and acceptability of emotional competence tele-interventions designed for Mandarin-speaking children with DLD in Taiwan.

Multiple studies have indicated that social stories are an effective means to help children acquire emotional knowledge [21-23]. Social stories have been widely used in clinical practice for children with autism spectrum disorder (ASD) [24]; however, the application of these stories for children with DLD remain understudied. To the best of our knowledge, few studies have focused on children with pragmatic language impairments [25], language impairments [26], and pragmatic disorders with behavioral difficulties [24]. Among the key factors that contribute to the efficacy of a social story is the display of the protagonist’s emotional state and social interactions [27], along with labeling affective, physical, and perceptual changes with words describing the protagonist’s mental state. These labels help children understand that different experiences can fulfill the same function as long as these experiences are perceived as having the same meaning [28]. The use of visual aids such as pictures or videos can enhance the emotional representation of potential causes, subjective feelings, physiological responses, and cognition during social interactions [29]. Thought bubbles can also be used to clarify the protagonist’s emotional state and thoughts within the social context [30,31]. Hence, emotional competence interventions conducted using social stories through remote interfaces, also referred to as social story tele-interventions (SSTIs), are expected to enhance the emotional competence of children with DLD.

### Objectives

Preschool and early elementary school are critical periods in the development of a child’s emotional competence; the child learns about self-regulation, and their level of emotional competence determines the severity and likelihood of behavioral problems [32,33]. In this research, we examined children in kindergarten and early elementary school. A total of 2 studies were conducted to explore 3 research questions. In study 1, our goal was to determine whether Mandarin-speaking children with DLD in Taiwan differ from those with TLD in emotional competence. We compared Mandarin-speaking children with DLD to those with TLD in terms of the size and depth of their understanding of emotional terms at the ages of 5 to 7 years. We hypothesized that Mandarin-speaking children aged 5 to 7 years with DLD would exhibit lower performance in the size and depth of their understanding of emotional terms compared with their counterparts with TLD even with family language characteristics such as maternal educational level accounted for. In study 2, we used an interrupted time-series design to explore two questions: (1) Do SSTIs enhance the understanding of emotional language by Mandarin-speaking children aged 5 to 7 years with DLD relative to their baseline performance (ie, before the intervention)? (2) Are SSTIs a feasible and acceptable intervention for these children in Taiwan? We hypothesized that SSTIs would enhance the emotional competence of Mandarin-speaking children aged 5 to 7 years with DLD and that this tele-intervention would be both feasible and acceptable for them and their parents in Taiwan.

### Study 1

#### Methods

This case-control study was conducted to compare the emotional competence of children with DLD to that of children with TLD.

#### Participants

**Figure 1:**
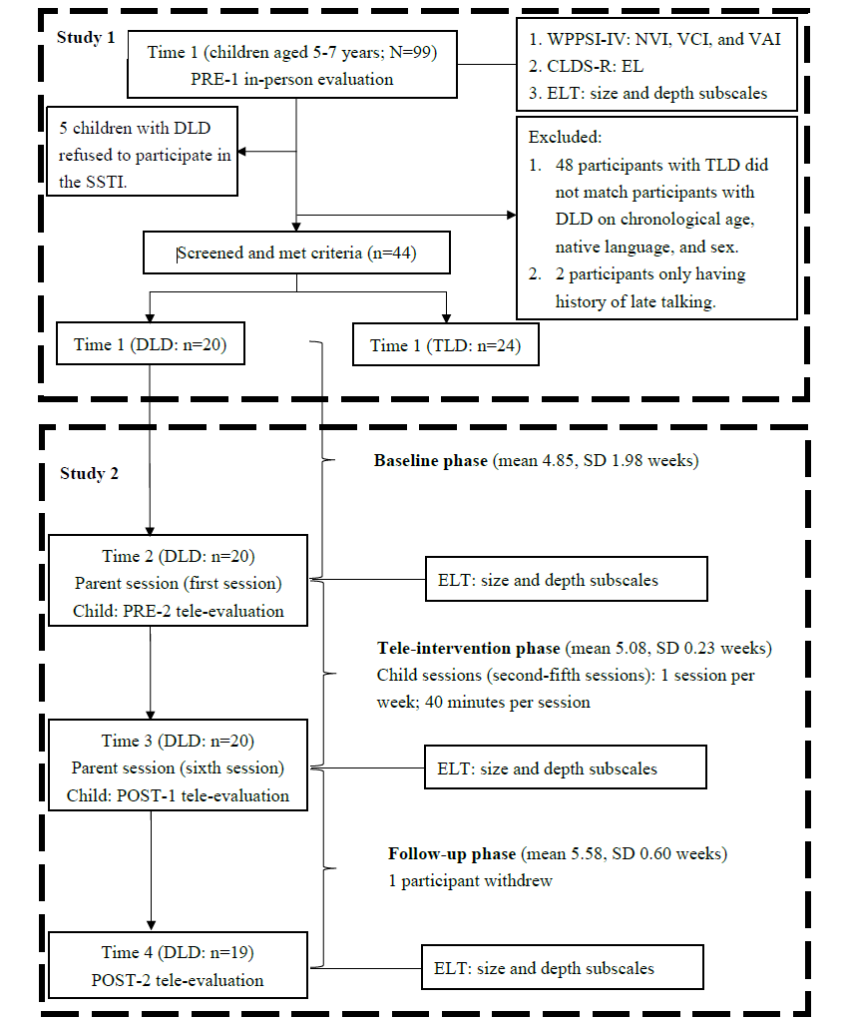
Flowchart of study 1 and study 2. CLDS-R: Child Language Disorder Scale–Revised; DLD: developmental language disorder; EL: expressive language; ELT: Emotional Lexicon Test; NVI: Nonverbal Index; POST-1: postintervention time point 1; POST-2: postintervention time point 2; PRE-1: preintervention time point 1; PRE-2: preintervention time point 2; SSTI: social story tele-intervention; TLD: typical language development; VAI: Vocabulary Acquisition Index; VCI: Verbal Comprehension Index; WPPSI-IV: Wechsler Preschool and Primary Scale of Intelligence, Fourth Edition.

A total of 99 children aged 5 to 7 years were recruited from parenting websites and local pediatric clinics in northern and central Taiwan. Figure 1 shows the process of recruitment in study 1. Children with DLD meeting the following matching criteria were included in the study: being within 3 months of the specified age range, having Mandarin Chinese as their native language, and being of the same sex as their matched counterparts. If >1 participant with TLD met these criteria and could be matched with a participant with DLD, they were all included in the analysis. A total of 48 participants with TLD who did not meet the aforementioned criteria and who completed only the in-person evaluation at the preintervention 1 time point (time 1) were excluded from the final analysis. In addition, 2 participants with only a history of late talking who also completed only the in-person evaluation at the preintervention 1 time point (time 1) were excluded from the final analysis. A total of 5 parents refused to participate in the subsequent tele-intervention program. Therefore, of a total of 25 parents of children with DLD, only 20 (80%) participated. Furthermore, ultimately, 44 children were included in the final analysis in study 1: 20 (45%) in the DLD group (mean age 5.79, SD 0.47 years) and 24 (55%) in the TLD group (mean age 5.93, SD 0.31 years).

All participants with DLD had received a diagnosis based on the *DSM-5* criteria at the Early Developmental Evaluation Center, the official institution designated by the Ministry of Health and Welfare of Taiwan for identifying and evaluating children with developmental disorders before the age of 7 years. In Taiwan, the gold standard for diagnosing DLD requires confirmation through a converging evidence approach [34], which includes the assessment of delays in receptive and expressive language by board-certified speech and language pathologists using parental reports, standardized tests, and clinical observations. Clinical psychologists are required to confirm the absence of cognitive delays, and child psychiatrists and pediatric neurologists are required to determine whether any observed language delays are due to other neurodevelopmental (eg, ASD) or neurological (eg, epilepsy) disorders. Establishing a DLD diagnosis through a converging evidence approach in clinical assessments and decision-making scenarios by medical professionals should be considered the gold standard. Participants with TLD had no known developmental or cognitive impairments.

#### Measurement

##### Family Characteristics

Data on family characteristics, including paternal and maternal mean age, paternal educational level and marital status, monthly family income, and number of siblings of the child participant, were collected from parent reports.

##### Child Characteristics

Data on child characteristics, including age, sex, developmental risk factors, and medical diagnoses, were also collected from parent reports. To confirm that the children met the inclusion criteria, their parents provided an early intervention assessment report issued by the Ministry of Health and Welfare. Among the developmental risk factors considered in this study were biological factors (eg, premature birth, low birth weight, genetic disorders, perinatal complications, and neurological conditions) and medical factors (eg, chronic diseases, infectious diseases, and hearing or vision impairments).

##### Evaluation of Nonverbal Ability

Nonverbal ability was measured using the Mandarin Chinese version of the Nonverbal Index (NVI) of the Wechsler Preschool and Primary Scale of Intelligence, Fourth Edition [35]. The NVI has an internal consistency reliability of 0.94 to 0.96 across various age groups and a test-retest reliability of 0.85 [35].

##### Evaluation of Language Abilities

A total of 3 dimensions of language abilities were evaluated: verbal comprehension, vocabulary acquisition, and linguistic expressivity. Verbal comprehension and vocabulary acquisition were evaluated using the Mandarin Chinese versions of the Verbal Comprehension Index (VCI) and Vocabulary Acquisition Index (VAI), respectively, of the Wechsler Preschool and Primary Scale of Intelligence, Fourth Edition [35]. The VCI and VAI have internal consistency reliability levels of 0.92 to 0.94 and 0.84 to 0.92, respectively, and both have a test-retest reliability of 0.86 [35]. Expressive language ability was evaluated using the Mandarin Chinese version of the Child Language Disorder Scale–Revised (CLDS-R) [36]. This subscale evaluates expressive language skills across multiple domains. These domains include (1) the ability to retrieve vocabulary or construct short phrases or sentences in response to questions that evaluate knowledge (eg, functions, general information, and analogies) acquired in the classroom and from one’s lived environment; (2) phonological working memory, measured through sentence repetition tasks in which children verbally recall sentences of increasing length and grammatical complexity; and (3) narrative skills, evaluated through spontaneous image description and story retelling tasks with or without the aid of sequenced images. The internal consistency reliability of each CLDS-R subscale across different age groups ranges from 0.80 to 0.96, with a test-retest reliability ranging from 0.92 to 0.98 [36].

##### Evaluation of Emotional Competence

Emotional competence was evaluated using 2 subscales from the Emotional Lexicon Test (ELT) [37]: a depth subscale and a size subscale. Illustrations were drawn without displaying the facial expression of the protagonist to avoid influencing the children’s responses. The depth subscale was used to evaluate the depth of understanding of basic emotional terms (BETs) and complex emotional terms (CETs). After the examiner read a story, they asked each child to label the emotional state of the protagonist and state the reasons underlying their responses (ie, explain the reasons underlying the protagonist’s feelings in this context). This subscale evaluates the ability of children to retrieve emotional terms through a recall mechanism. Responses are rated depending on their quality on a scale with end points from 0 to 2, reflecting the varying degrees of children’s understanding of emotional terms. In terms of interrater reliability, this subscale was evaluated by 2 raters, who reported a 95% agreement. Discrepancies in scores were discussed by the 2 raters to reach a consensus. The size subscale was used to evaluate the size of BETs and CETs. After the examiner read a story, they asked each child to determine which of 2 emotional state terms (eg, joyful vs fearful, scared vs ashamed, and delighted vs sad) better described the protagonist’s emotional state. This subscale evaluates the ability of children to recognize emotional terms through a recognition mechanism. Each correct recognition response is assigned a score of 1 point, indicating the size of the child’s emotional lexicon. These 2 subscales have been adopted in other emotional lexicon tests [38]. Each subscale consists of 14 cards with short illustrated stories, of which 6 feature basic emotions (joy, sadness, happiness, anger, fear, and disgust) and 8 feature complex emotions (shame, contempt, guilt, hate, envy, jealousy, pride, and loneliness). Hence, the scores on the depth subscale range from 0 to 28 points, and the scores on the size subscale range from 0 to 14 points. Each item in the ELT includes images to convey the story content, which in turn reduces the semantic comprehension load of the participating child. In this study, before the formal items were used, 2 questions and 1 example item were presented to determine whether the children could match the images with the oral content, indicating their understanding of the story. ELT has an adequate to high internal consistency (0.70-0.71) for CETs [39]. Its criterion-related validity scores with the Metacognitive Vocabulary Test and Peabody Picture Vocabulary Test–Revised are 0.80 and 0.74, respectively [40]. In addition, both hard- and soft-copy versions of the ELT were used. The hard-copy version was used for in-person evaluations, whereas the soft-copy version was used for tele-evaluations.

#### Procedure

After the children arrived at the laboratory with their parents, all parents were informed of the research procedures. All children successfully completed the 2 questions and 1 example item before the administration of the ELT proper, indicating their understanding of the story. All tests were conducted by licensed clinical psychologists and graduate students trained in child psychological assessment.

#### Ethical Considerations

The study design was approved by the institutional review board of Chung Shan Medical University Hospital in Taiwan (CS2-19046). During the in-person evaluation phase at the preintervention 1 time point (time 1), the researchers outlined the following: (1) study purpose, (2) data handling procedures, (3) privacy measures, and (4) participant rights, including the voluntary nature of participation and withdrawal options. All parents understood and provided their informed consent. This study was conducted in accordance with relevant guidelines and regulations for human participants. Privacy measures included secure data storage with access restricted to research team members only.

#### Data Analysis

Separate analyses of covariance (ANCOVAs) were conducted to determine the differences in the NVI, VCI, VAI, and linguistic expressivity scores between the 2 groups, with family characteristics used as a covariate. Chi-square and Fisher exact tests were used to compare group proportions with qualitative data for family characteristics (ie, parental educational level and marital status, monthly family income, and whether the child participant had siblings) and child characteristics (ie, sex and presence of developmental risk factors). In addition, a 3-way mixed-model multivariate ANCOVA was conducted, with group (TLD vs DLD) used as the between-group independent variable and emotion (basic vs complex) and understanding (size vs depth) used as the within-group independent variables, with maternal educational level used as a covariate. In case a statistically significant 3-way interaction was observed among group, emotion, and understanding, the simple main effects between TLD and DLD were examined. Effect sizes from ANCOVAs were calculated using the partial η_p_^2^, which could be directly translated into a percentage of explained variance. Using the basic framework by Cohen [41], we interpreted these effect sizes as small (η_p_^2^=0.01), moderate (η_p_^2^=0.06), or large (η_p_^2^=0.15). We also conducted a post hoc power analysis for ANCOVAs using G*Power (version 3.1.5) for a sample size of 44 participants, with α=.05 [42]. Post hoc power values of >0.8, between 0.6 and 0.8, and <0.6 were considered high, moderate, and low, respectively. All statistical analyses were conducted using SPSS Statistics (version 25.0; IBM Corp).

#### Results

##### Participant Characteristics

Table 1 presents the characteristics of the children and their parents in the TLD and DLD groups. Compared with the TLD group, the DLD group had a lower maternal educational level (N=44, χ^2^_1_=5.4, *P*=.04). Previous studies have found that mothers’ educational level is related to children’s social, emotional, and academic development [43]. Therefore, we controlled for this variable when conducting the ANCOVAs. However, no differences were observed between the 2 groups (*P*>.05) in terms of child characteristics (ie, mean age, sex, and risk factors) and family characteristics (ie, paternal and maternal mean age, paternal educational level and marital status, monthly family income, and whether the child participant had siblings).

Table 2 presents the NVI, VCI, VAI, and oral expression scores of the TLD and DLD groups during the evaluation at the preintervention 1 time point. No significant difference was observed in the NVI between the 2 groups (*P*>.05), as indicated by the ANCOVA results. However, compared with the TLD group, the DLD group exhibited significantly lower levels of verbal comprehension, vocabulary acquisition, and linguistic expressivity (*F_1, 41_*=17.20, 11.89, and 10.91, respectively; *P*<.001, *P*=.001, and *P*=.002, respectively; η_p_^2^=0.30, 0.23, and 0.21, respectively; power=0.99, 0.94, and 0.92, respectively).

**Table 1:** Characteristics of the children and their parents.a

Variable				TLD^b^ (n=24)		DLD^c^ (n=20)		*P* value	
Child’s age (y), mean (SD)				5.93 (0.31)		5.79 (0.47)		.26	
Father’s age (y), mean (SD)				40.58 (4.72)		42.05 (5.16)		.34	
Mother’s age (y), mean (SD)				37.54 (3.75)		39.65 (4.20)		.09	
**Child characteristics, n (%)**									
	**Sex**								.76
		Male	17 (71)		15 (75)				
		Female	7 (29)		5 (25)				
	**Developmental risk factors**								.15
		Yes	13 (54)		15 (75)				
		No	11 (46)		5 (25)				
**Family characteristics, n (%)**									
	**Father’s educational level**								>.99
		Below university level	3 (12)		3 (15)				
		At or above university level	21 (88)		17 (85)				
	**Mother’s educational level**								.04
		Below university level	1 (4)		6 (30)				
		At or above university level	23 (96)		14 (70)				
	**Parents married and living together**								>.99
		Yes	22 (92)		17 (85)				
		No	2 (8)		3 (15)				
	**Monthly family income**								.14
		<NT $^d^ 59,999 (US $1821.35)	10 (42)		13 (65)				
		≥NT $ 60,000 (US $1821.38)	14 (58)		7 (35)				
	**Child participants having siblings**								.50
		Yes	8 (33)		4 (20)				
		No	16 (67)		16 (80)				

^a^All *P* values were obtained from ANOVAs or chi-square or Fisher exact tests.

^b^TLD: typical language development.

^c^DLD: developmental language disorder.

**Table 2:** Nonverbal and language abilities of the participants^a^.

Variable	TLD^b^ (n=24), mean (SD)	DLD^c^ (n=20), mean (SD)	*P* value
NVI^d^ (CS^e^)	103.71 (9.14)	98.15 (14.79)	.23
VCI^f^ (CS)	112.83 (12.67)	93.65 (15.52)	<.001
VAI^g^ (CS)	111.63 (11.21)	96.55 (13.92)	.001
EL^h^ (*z* score)^i^	−0.08 (0.90)	−0.97 (0.61)	.002

^a^All *P* values were obtained from analyses of covariance.

^b^TLD: typical language development.

^c^DLD: developmental language disorder.

^d^NVI: Nonverbal Index.

^e^CS: composite score of the Wechsler Preschool and Primary Scale of Intelligence, Fourth Edition, Chinese version.

^f^VCI: Verbal Comprehension Index.

^g^VAI: Vocabulary Acquisition Index.

^h^EL: expressive language.

^i^*z* score of the Child Language Disorder Scale–Revised, Chinese version.

##### Emotional Competence Among Children With TLD and DLD

**Figure 2:**
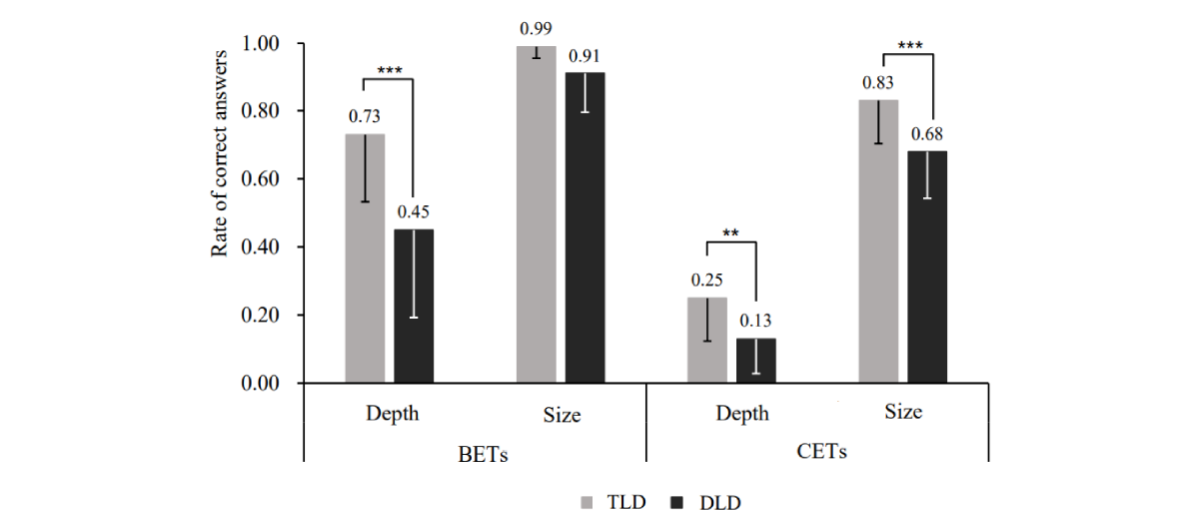
Mean rate of correct answers for basic emotional terms (BETs) and complex emotional terms (CETs) for the typical language development (TLD) and developmental language disorder (DLD) groups during the evaluation at preintervention time point 1 (time 1). The vertical lines represent error bars with 1 SD. The asterisks indicate the significant simple main effects of group, revealing a higher mean rate of correct answers for the TLD group than for the DLD group. **P<.01; ***P<.001.

Figure 2 illustrates the size and depth of understanding of BETs and CETs for the TLD and DLD groups during the evaluation at the preintervention 1 time point. It also shows the main effects of emotion (Wilks λ_1, 41_=34.17; *P*<.001; η_p_^2^=0.45; power=0.99) and understanding (Wilks λ_1, 41_=72.97; *P*<.001; η_p_^2^=0.64; power=0.99). A post hoc analysis revealed that BETs outperformed CETs (*P*<.001), with the number of recognized emotional terms being greater than that of recalled emotional terms (*P*<.001). A statistically significant 3-way interaction was observed among group, emotion, and understanding (Wilks λ_1, 41_=12.39; *P*=.001; η_p_^2^=0.23; power=0.66). Simple-simple main effect analysis revealed that the DLD group scored lower than the TLD group in terms of the depth of understanding of BETs and the size and depth of understanding of CETs (*F_1, 164_*=37.20, 11.48, and 6.54, respectively; *P*<.001, *P*<.001, and *P*=.01, respectively; η_p_^2^=0.18, 0.07, and 0.04, respectively; power=0.86, 0.43, and 0.26, respectively).

#### Discussion

##### Principal Findings

Few studies have compared the emotional competence of Mandarin-speaking children with DLD to that of children with TLD. In this study, we controlled for child and family characteristics in our research design and statistical analysis while comparing children with DLD and TLD. This approach increased the validity of our findings, indicating a lag between children with DLD and those with TLD in terms of emotional competence. It also enhanced the validity of study 2, which examined the improvement observed in emotional competence among children with DLD after undergoing a tele-intervention.

The size of understanding of BETs among children aged 5 to 7 years with DLD was similar to that among their counterparts with TLD, indicating that children with DLD can understand basic emotional states in typical social scenarios. However, the depth of understanding of BETs among children aged 5 to 7 years with DLD was considerably lower than that among their counterparts with TLD, indicating that the ability of children with DLD to express their emotions through BETs may be limited. In addition, children with DLD were slower in understanding CETs compared with children with TLD in kindergarten and early elementary school years, indicating the delayed development and mastery of such concepts. Overall, our findings are consistent with those of previous studies [5-7,14,44] indicating that Mandarin-speaking children with DLD also exhibit impaired emotional competence. These findings suggest that the lag observed in emotional competence in children with DLD is a universal characteristic across countries and ethnic groups [45]. These findings underscore the importance of early interventions in improving their conceptual knowledge of emotional states.

In this study, children with DLD exhibited language functioning within the low but normal range, although their scores were lower than those of their peers with TLD. The study explored whether these children experienced delays in their lexical, grammatical, or articulatory development. In study 1, all children had been diagnosed with DLD in accordance with *DSM-5* criteria at the Early Developmental Evaluation Center, an institution designated by the Ministry of Health and Welfare of Taiwan to evaluate developmental disorders in children aged <7 years. Diagnosing DLD through clinical assessments with a converging evidence approach for clinical decision-making is regarded as the gold standard in Taiwan. In this approach, no single method serves as the deciding factor in making diagnostic decisions regarding the receptive and expressive language skills of individuals with DLD. Converging evidence refers to the concept that multiple pieces of assessment data must align and point in the same direction to support a diagnostic decision [34]. In specific test areas, the cutoff score may vary to maximize sensitivity and specificity in identifying those who are at a high risk of developmental problems. Nevertheless, no consensus has been reached regarding the cutoff values for the VCI, the VAI, and the CLDS-R expressive language subscale required for determining whether a child has DLD. In this study, the VCI, the VAI, and the CLDS-R expressive language subscale were used only to evaluate the language capabilities of children with DLD and compare them to those of children with TLD. Our goal was to determine whether the language capabilities of children with DLD lagged behind those of children with TLD, unlike nonverbal capabilities, for which no differences were observed between the 2 groups. Notably, we did not use the VCI, the VAI, or the CLDS-R expressive language subscale for diagnostic purposes in this study.

According to Bishop [46], DLD involves heterogeneous language features. In this study, we examined only the verbal comprehension, vocabulary acquisition, and expressive language capabilities of children with DLD and did not evaluate their speech perception, phonological awareness, or understanding and use of grammar. Therefore, we could not confirm whether children with DLD lag in these language dimensions. In addition, no consensus has yet been reached regarding the definition of DLD in terms of whether receptive or expressive language should be scored at a threshold of <1 or 1.5 SDs in standardized language tests [47]. Of a total of 20 children with DLD, 6 (30%) had received physical therapy, 19 (95%) had received occupational therapy, 18 (90%) had received speech therapy, and 6 (30%) had received psychological therapy, indicating that these children continually underwent systematic and structured interventions implemented by medical professionals to optimize their development during sensitive periods [48-50]. Therefore, although DLD can be qualitatively defined in accordance with the *DSM-5* [1] in medical and research contexts, no consensus has yet been established regarding its quantitative definition, including which tests to conduct and which cutoff criteria to apply. Furthermore, each quantitative definition may have unique implications for specific contexts in different countries or cultures, presumably reflecting the use of different measurement tools and cultural perspectives on children’s development and delays [51]. Bishop [46] emphasized the importance of establishing a consensus regarding the quantitative definition of DLD to facilitate cross-linguistic and cross-cultural comparisons of the characteristics, developmental changes, and emotional competence of children with DLD over time.

##### Conclusions

In this study, low power values were observed for the simple-simple main effects of the size and depth of understanding of CETs. According to Ryou et al [52], a lower power value indicates a higher risk of a type II error, also referred to as a false negative, in which a statistical test does not call for the rejection of the null hypothesis when the alternative hypothesis is true. However, our group comparisons for the size and depth of understanding of CETs were significant, with moderate effect sizes. These comparisons mitigated the risk of type II errors in comparison to scenarios in which group differences were not significant. Therefore, increasing the sample size in future studies may aid in achieving more robust results.

### Study 2

#### Methods

In this study, we used a prospective 1-group interrupted time-series research design situated within the context of a response-to-intervention instructional framework [53]. This study comprised a baseline phase, a tele-intervention phase, and a follow-up phase.

#### Participants

The same 20 children with DLD who participated in study 1 were included in study 2.

#### SSTI Characteristics

##### Intervention Sessions

This 6-week emotional competence tele-intervention comprised 2 sessions for parents (first and sixth sessions) and 4 sessions for children (second to fifth sessions). In the first parent session, the mechanism of SSTIs designed for children to acquire emotional competence skills was introduced, whereas in the last parent session, the SSTIs and children’s emotional competence skills acquired during the sessions and on a daily basis were reviewed. No home practice was offered after the 2 parent sessions, and no children were involved in these sessions. Each child’s session included the presence of their parents and comprised the following elements: (1) a parent-child interactive game, that is, the finger trap game; (2) a 4-panel physical story comic; (3) a social story video (Multimedia Appendix 1) played for the participating child 3 times; and (4) a talk practice (retelling, prompting, self-narration, and imitation).

In the finger trap game, each parent places their palm facing down, and each child places their index finger underneath their parent’s palm. Once a specific word (eg, *three*) is presented in a series of words (eg, *three*, *four*, *six*, *two*, *three*...), the parent attempts to rapidly close their open palm to catch the child’s finger. Two 4-panel physical story comics are presented. The first comic, used in the first and second child sessions, depicts a balloon being blown away by the wind, floating up into a tree, getting caught on a branch, and popping. The second comic, used in the third and fourth child sessions, depicts a clear, sunny day that later turns cloudy and then rains heavily.

The themes and language content of these intervention sessions corresponded to the titles of 4 researcher-edited social story videos, namely, “Great with others,” “Play game,” “Grab a toy,” and “Give a gift.” In these social story videos, external causes lead the protagonist to have an emotional experience. The social stories developed in this study included 4 types of mental state terms: cognitive state terms such as *know*; desire state terms such as *want*; perceptual state terms such as *feel*; and basic emotional state terms such as *happy*, *sad*, *angry*, and *fear* [54]. Table 3 presents the mental state terms and their frequencies in each social story. Thought bubbles are used to display the beliefs, desires, intentions, knowledge, and moods of the story’s protagonist as they arise from the interpretation of environmental cues (Multimedia Appendix 1). As shown in Multimedia Appendix 1, panel 2 displays the mental state for desire, panels 4 and 5 display the mental state for belief, panel 7 displays the mental state for intention, and panels 3 and 6 display the mental states for moods associated with desire and belief.

**Table 3:** Mental state terms and frequency, words, and length in each social story.

Social story	Mental state terms (frequency)	Words, N^a^	Length (seconds)
Great with others	Know (n=6) Want (n=6) Feel (n=6) Happy (n=6) Sad (n=6)	115	95
Play game	Know (n=6) Want (n=6) Feel (n=6) Happy (n=6) Angry (n=6)	118	104
Grab a toy	Know (n=6) Want (n=6) Feel (n=6) Happy (n=6) Fear (n=6)	112	93
Give a gift	Know (n=3) Want (n=3) Feel (n=3) Happy (n=3) Sad (n=3) Angry (n=3) Fear (n=3)	101	91

^a^Chinese character count.

The following instructions were given to the children for the talking practice. For retelling, the instruction was as follows: “Tell me about the story you just heard.” For prompting, the instruction was as follows: “I have a few questions to ask you about this story.” For self-narration, the instruction was as follows: “After listening to this story, it’s your turn to share a story about how you grabbed a toy with someone.” For imitation, the instruction was as follows: “Tell me the story one more time—I’ll say a sentence, and then you repeat it.”

Each session lasted approximately 40 minutes. A total of 4 sessions were delivered over 4 weeks, with 1 session per week. The quantitative and qualitative aspects of this intervention program are consistent with those outlined by Frizelle et al [55], who emphasized that, in an intervention program, the concept of efficiency is central to “dosage,” which includes both quantitative (number of sessions, frequency, and duration) and qualitative (form) constructs.

#### Tele-Intervention Information and Communications Technology

The technical setup used by the 3 interventionists in their workrooms included a laptop equipped with Google Meet, a broadband internet connection, a web camera, a flexible lighting device to achieve optimal lighting, and a backdrop of an image showing the name of the interventionist to ensure optimal visibility during the tele-intervention sessions. The technical setup used by the participants included their own personal desktop computer or laptop (9/20, 45%), tablet (3/20, 15%), or smartphone (8/20, 40%), all equipped with Google Meet; an internet connection; and a web camera. All parents reported having internet access at their homes (fixed line or 4G or 5G cellular internet). They also reported a sound level of 55 to 65 dB, measured using a sound meter app. Throughout the tele-intervention sessions, the participants were asked to keep the video call in full-screen mode. All interventionists used the screen-sharing tool to present visual stimuli for the social story video and prompt pictures during the talk practice stage.

#### Measurement

##### Assessment of Emotional Competence

In this study, the ELT was used to evaluate the emotional competence of children with DLD. The study 1 section provides more details regarding this test.

##### Assessment of the Feasibility and Acceptability of SSTIs

Feasibility was evaluated based on recruitment, retention, and attendance rates to each session, and acceptability was evaluated based on the outcomes of each session. The participants were asked to either select 1 of 4 affect categories (happiness, anger, fear, and sadness) or remain neutral. Reports of happiness and neutrality were considered acceptable.

#### Procedure

As shown in Figure 1, the DLD group completed the in-person evaluation at the preintervention 1 time point (time 1). After 5 to 6 weeks, the SSTIs were administered. The parents attended the first parent session, and the ELT was administered during the tele-evaluation at the preintervention 2 time point (time 2). After 1 week, the DLD group received the SSTIs, which lasted 4 weeks (second to fifth session). In the sixth session, the parents were evaluated, and the DLD group was administered the ELT again during the tele-evaluation at the postintervention 1 time point (time 3). Finally, 5 to 6 weeks after time 3, the DLD group was administered the ELT once again during the tele-evaluation at the postintervention 2 time point (time 4). Only 1 participant withdrew from the study during the tele-evaluation at the postintervention 2 time point. The mean number of weeks between time 1 and time 2 and between time 3 and time 4 was similar (*P*>.05). Figure 1 shows the participant recruitment process. The period from time 1 to time 4 spanned approximately 18 to 20 weeks for each participant with DLD.

#### Ethical Considerations

This study was conducted in accordance with relevant guidelines and regulations for human participants. Privacy measures included secure data storage with access restricted to research team members only. The study design was approved by the institutional review board of Chung Shan Medical University Hospital in Taiwan (CS2-19046).

#### Data Analysis

When the data suggest a nonlinear relationship, piecewise linear growth models can be used to divide growth trajectories into >2 linear components, such as in interrupted time-series data [56,57]. In this study, we used a piecewise linear growth model with 3 slopes for each outcome: slope 1 to evaluate changes from the preintervention 1 to preintervention 2 time points (baseline), slope 2 to evaluate changes from the preintervention 2 to postintervention 1 time points (tele-intervention), and slope 3 to evaluate changes from the postintervention 1 to postintervention 2 time points (follow-up). We also hypothesized that the tele-interventions would have an effect. Generally, the absence of an intervention effect for baseline and follow-up indicates that emotional competence did not increase during the baseline phase and did not decrease during the follow-up phase. The HLM software (version 7.03; Scientific Software International) [58] was used to fit the piecewise linear growth model. All other analyses were conducted using SPSS Statistics (version 25.0). Estimates of intraclass correlation coefficients (ICCs) are often used to analyze the power of piecewise linear growth models [59]. These coefficients measure the similarity between data points within the same cluster and those in different clusters. They typically range from 0 (no similarity between clusters) to 1 (complete similarity between clusters). ICCs of >0.20 indicate that the model can detect actual differences in growth rates across different time segments [60]. Sufficient power is essential for study accuracy, making piecewise linear growth models suitable for analysis. Effect sizes are typically calculated for each period using the Cohen *d* for repeated measures [61]. Values of 0.2, 0.5, and 0.8 indicate small, moderate, and large effects, respectively [62]. The Cohen *d* was calculated using G*Power (version 3.1.5) [63].

#### Results

##### Changes in Emotional Competence Among Participants With DLD

**Figure 3:**
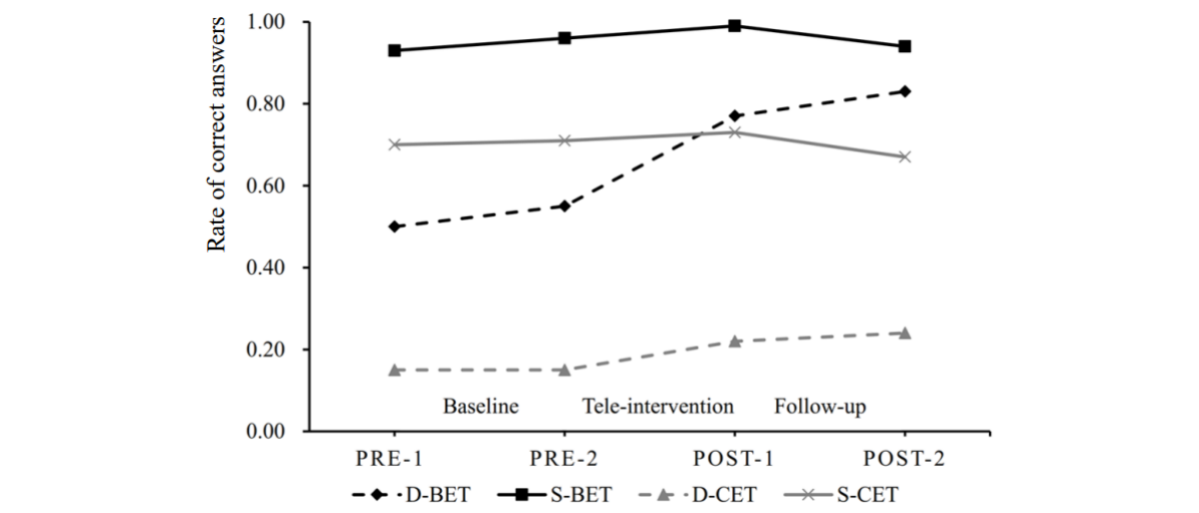
Mean change trajectories for the size and depth of basic emotional terms (BETs) and complex emotional terms (CETs) during the baseline, tele-intervention, and follow-up phases. D-BET: BET depth; D-CET: CET depth; POST-1: postintervention time point 1; POST-2: postintervention time point 2; PRE-1: preintervention time point 1; PRE-2: preintervention time point 2; S-BET: BET size; S-CET: CET size.

In this study, the ICCs for BET size, BET depth, CET size, and CET depth were <0.001, 0.34, 0.30, and 0.46, respectively. The ICCs for BET depth, CET size, and CET depth were >0.20, indicating that the piecewise linear growth model was suitable for analysis. Figure 3 shows the mean change trajectories for the size and depth of BETs and CETs. Table 4 presents the results of multilevel piecewise growth models for estimating mean changes in 4 emotion outcomes in the tele-intervention during the baseline (preintervention 1 to preintervention 2 time points), tele-intervention (preintervention 2 to postintervention 1 time points), and follow-up (postintervention 1 to postintervention 2 time points) phases. Regarding BET size, no statistically significant change was observed during the baseline, tele-intervention, or follow-up phase (*P*>.05). Regarding BET depth, the rate of correct answers significantly increased during the tele-intervention phase (*P*<.001), but no significant change was observed during the baseline or follow-up stage (*P*>.05). Regarding CET size, no statistically significant change was observed during any phase (*P*>.05). Regarding CET depth, the rate of correct answers significantly increased during the tele-intervention phase (*P*<.001), but no significant change was observed during the baseline or follow-up phase (*P*>.05).

**Table 4:** Baseline, tele-intervention, and follow-up changes in multilevel piecewise models for emotional terms.

Variable			Size of BET^a^, B (95% CI)^b^		Depth of BET, B (95% CI)^c^	Size of CET^d^, B (95% CI)^e^		Depth of CET, B (95% CI)^f^
**Fixed effect**								
	Intercept	0.93^g^ (0.89 to 0.98)		0.50^g^ (0.40 to 0.61)		0.69^g^ (0.63 to 0.76)	0.15^g^ (0.10 to 0.19)	
	Baseline	0.03 (–0.04 to 0.09)		0.05 (–0.03 to 0.13)		0.01 (–0.05 to 0.07)	0.00 (–0.05 to 0.05)	
	Tele-intervention	0.03 (–0.01 to 0.08)		0.21^g^ (0.11 to 0.32)		0.03 (–0.05 to 0.10)	0.07^g^ (0.04 to 0.10)	
	Follow-up	−0.02 (–0.04 to 0.00)		0.05 (–0.05 to 0.14)		0.001 (–0.09 to 0.09)	0.01 (–0.04 to 0.06)	

^a^BET: basic emotional term.

^b^Random effect—intercept (U_0_): χ^2^_19_=15.4; *P*>.50.

^c^Random effect—intercept (U_0_): χ^2^_19_=116.3; *P*<.001.

^d^CET: complex emotional term.

^e^Random effect—intercept (U_0_): χ^2^_19_=54.2; *P*<.001.

^f^Random effect—intercept (U_0_): χ^2^_19_=98.6; *P*<.001.

^g^*P*<.001.

Table 5 presents the means and SDs of various emotional term measures at 4 measurement time points. The effect sizes for changes during the baseline period (from the preintervention 1 to preintervention 2 time points) were small (Cohen *d*=0.00-0.26), indicating that the depth and size of BETs and CETs slightly changed during this period. During the tele-intervention period (from the preintervention 2 to postintervention 1 time points), large effect sizes (Cohen *d*=0.88-0.94) were observed for the changes in depth of BETs and CETs, whereas small effect sizes (Cohen *d*=0.12-0.27) were observed for the changes in size of BETs and CETs. During the follow-up period (from the postintervention 1 to postintervention 2 time points), small effect sizes (Cohen *d*=0.01-0.39) were observed for the changes in depth and size of BETs and CETs.

**Table 5:** Means, SDs, and effect sizes for emotional term measures.

	Rate of correct answers (PRE-1^a^), mean (SD)	Rate of correct answers (PRE-2^b^), mean (SD)	Rate of correct answers (POST-1^c^), mean (SD)	Rate of correct answers (POST-2^d^), mean (SD)	Effect size, Cohen *d*		
					Baseline	Tele-intervention	Follow-up
D-BET^e^	0.50 (0.25)	0.55 (0.30)	0.77 (0.19)	0.81 (0.21)	0.26	0.88	0.18
S-BET^f^	0.93 (0.10)	0.96 (0.09)	0.99 (0.04)	0.97 (0.06)	0.19	0.27	0.39
D-CET^g^	0.15 (0.10)	0.15 (0.11)	0.22 (0.13)	0.22 (0.13)	0.00	0.94	0.03
S-CET^h^	0.69 (0.15)	0.71 (0.10)	0.73 (0.20)	0.73 (0.10)	0.14	0.12	0.01

^a^PRE-1: preintervention 1 time point.

^b^PRE-2: preintervention 2 time point.

^c^POST-1: postintervention 1 time point.

^d^POST-2: postintervention 2 time point.

^e^D-BET: depth of basic emotional term.

^f^S-BET: size of basic emotional term.

^g^D-CET: depth of complex emotional term.

^h^S-CET: size of complex emotional term.

#### Feasibility and Acceptability of SSTIs

Of the 25 parents of participants with DLD, 5 (20%) did not take part in this study. Therefore, the recruitment rate for the program was 80% (20/25). At the preintervention 2, postintervention 1, and postintervention 2 time points, the retention rates were 100% (20/20), 100% (20/20), and 95% (19/20), respectively. During the 4 sessions, the attendance rates were 100% (20/20), 100% (20/20), 95% (19/20), and 100% (20/20). All participants (20/20, 100%) completed the 4 tele-intervention sessions, with only 5% of missing data at the postintervention 2 time point.

During the 4 sessions, the percentages of participants who reported being happy were 90% (18/20), 79% (15/19), 85% (17/20), and 90% (18/20), and the percentages of participants who reported being neutral were 10% (2/20), 11% (2/19), 15% (3/20), and 5% (1/20). Overall, the acceptability rates were 100% (20/20), 89% (17/19), 100% (20/20), and 95% (19/20) during the 4 sessions.

#### Discussion

##### Principal Findings

Tele-interventions are not a novel concept in medicine. However, to the best of our knowledge, this is the first study to integrate a social story intervention with a remote format to improve the emotional competence of children with DLD. In this study, a 3-stage process was used to observe changes in emotional terms among children with DLD. We discovered that changes in emotional competence occurred only during the tele-intervention phase, with no changes observed during the baseline phase before the intervention. These effects remained during the follow-up period after the intervention. Overall, this research design enhanced the validity of the results.

Our results indicated that SSTIs helped children with DLD deepen their understanding of BETs. In these SSTIs, 4 BETs were repeatedly used in various social contexts to reflect different individuals’ mental states. This approach enabled the participants to understand that a single emotion word may be applicable to a range of scenarios (ie, unspecific use). The earlier these terms are integrated into the active vocabulary of children, the more their use aligns with that of adults. These findings are consistent with those of Grosse et al [64] indicating that children who learn words about emotions through cross-context scenarios can understand and use such vocabulary as adults. In this study, thought bubbles were used to enhance the children’s understanding of representational mental states associated with emotional terms.

Although our intervention was not designed to introduce new CETs, we hypothesized that children with DLD would not seek to increase the size of their CETs and would rather seek to improve their understanding of the CETs that they had already learned. Overall, our findings indicate that SSTIs, which are based on a thorough understanding of BETs, can help children with DLD gain a deeper understanding of the CETs that they have already learned. Further systematic studies of domain-specific vocabulary are required to determine the effectiveness of SSTIs, including the size of emotion vocabulary and the depth of understanding.

Although social stories are often applicable to patients with ASD, similar to the application of such stories to children with ASD, the social stories designed in this study included visualizations of the story context, which can influence social storytelling by reducing extraneous cognitive demands [65]. When social stories are used as an intervention for children with DLD, adjustments may be required depending on the children’s language levels. In this study, we used the thought bubbles corresponding to mental state words, which can also be used to clarify the protagonist’s emotional state and mental thoughts within a social context. In other words, by making hidden thoughts and feelings explicit, children with DLD can gain a deeper understanding of their own and others’ emotions and acquire the emotional language necessary to reflect upon and discuss these emotions [66]. Given that children with DLD represent a highly heterogeneous group, when social story interventions are implemented for these children, their language levels must be considered. In other words, the vocabulary, syntactic structures, and story elements included must be carefully planned to ensure that they are within the instructional level and not the frustration level [67]. These strategies have the potential to enhance the expressive language output of children for discussing emotional topics, processing targeted emotional components, and engaging in tele-interventions.

Among the indicators of feasibility for evaluating a pilot program are recruitment, retention, fidelity, acceptability, adherence, and engagement [68,69]. In this study, we evaluated the indicators of recruitment (proportion of parents who were willing to participate), retention (proportion of parents who did not withdraw), and attendance (proportion of parents who attended all 4 sessions). Retention rates are often used as evidence of feasibility in early intervention studies [70,71], particularly for pilot programs [69]. A high retention rate indicates that the program has promise for further development. Future research is required to evaluate the indicators of fidelity, adherence, and engagement to strengthen feasibility assessments.

According to Gartlehner et al [72], clinicians and policy makers tend to differentiate between efficacy and effectiveness. These 2 constructs exist on a continuum where efficacy and efficiency are about whether an intervention functions well under ideal and real-world conditions, respectively. This study was conducted under ideal conditions, supervised by a clinical researcher with expertise in children with DLD. The sample size was small, and the participants were carefully selected—only children with a confirmed diagnosis of DLD were included. The outcomes focused on specific aspects of emotional competence, such as the acquisition of emotional terms, rather than on broader health measures. A short-term intervention was implemented to provide initial evidence of efficacy, with strict protocols for both care providers and families. Future studies should explore the effectiveness of this program for Mandarin-speaking children with DLD, but first, it is necessary to establish the importance of continually evaluating its intermediate- and long-term efficacy. Chorpita et al [73] have argued that, even if >10 successful replications support a particular treatment, this information would not be sufficient to determine whether this treatment is more suitable for a particular child than a treatment supported by only 2 instances of replication assuming the absence of predictors of differences in outcomes. This assumption underscores the importance of conducting effectiveness studies to determine whether intervention programs can be widely implemented in clinical settings. Therefore, future studies should examine our program’s real-world effects in hospital settings, including its acceptability, feasibility, and cost versus benefit. They should also explore broader mental health outcomes, such as increased self-regulation or decreased behavioral problems, of tele-interventions designed for Mandarin-speaking children with DLD.

Some parents raised concerns regarding their children facing difficulties focusing on their lessons and interacting with their instructors on-screen. Generally, in-person interventions involve physical contact and social interactions, such as high fives, contingency, eye contact, and joint attention, which can help children focus. In contrast, web-based interactions involve only visual support and behavioral management strategies to keep children engaged [74-76]. Despite these limitations, we discovered that the parents and children who participated in the intervention had high attendance rates and reported positive emotions after the intervention, indicating the feasibility and acceptability of SSTIs among them.

In the past few years, many hospitals were closed to the public because of the COVID-19 pandemic, thus preventing the implementation of in-person interventions. Early interventions for children with developmental delays were also suspended. These measures resulted in an increase in tele-evaluations and tele-interventions in medical scenarios. Although specialized hospitals are currently available for children, tele-practice interventions are particularly valuable for families living in rural areas, who tend to face challenges such as time constraints and lack of transportation. In these areas, enhancing the adoption of tele-practice requires software and hardware support because of these areas’ limited internet access and lack of hardware [77]. Currently, Taiwan is striving to expand its 5G infrastructure (through platforms such as OpenGov Asia, the Ministry of Digital Affairs, and ComSoc Technology), particularly in remote areas such as Penghu, to improve digital accessibility and support tele-practice. Enhancing the degree of acceptance among health care professionals and parents is essential for promoting child language development and raising awareness of the benefits of tele-practice [78,79]. Finally, tele-interventions can also be used as a hybrid model in conjunction with in-person services to enhance learning experiences and make efficient use of resources.

This study has some limitations. First, emotional competence was exclusively measured by determining the number of emotional terms that children with DLD were aware of and examining the depth of their understanding of these terms. These measures did not reflect whether the children’s emotional regulation strategies or behavioral problems improved as a result of our intervention. Therefore, further studies are required to explore these aspects of emotional competence. Second, the period during which we evaluated the effects of the intervention was short. Therefore, further long-term follow-up studies are required to determine the long-term and cumulative effects of our tele-intervention. Third, although the Italian version of the ELT has demonstrated strong psychometric characteristics [40] and assesses both basic and complex emotional competence in children, the Chinese version of this test lacks support from psychometric data. As in previous studies [38], we evaluated children’s understanding of emotion-related words by making them listen to stories and choose the correct terms to determine the quantity of emotion words. We subsequently asked them to explain their choices to gauge their depth of understanding. This approach, which relies on a single test, limits the concept of emotional competence in children. Therefore, future studies should involve various additional measures such as the Emotional Competencies Scale for Young Children [80] or collect data through parent questionnaires or transcript analyses to determine whether children use emotion words in different contexts [81,82].

##### Conclusions

Mandarin-speaking children aged 5 to 7 years with DLD exhibit lower emotional competence than those with TLD even after adjusting for child and family language characteristics. Although DLD can be qualitatively defined in accordance with the *DSM-5* [1] in medical and research contexts, no consensus has yet been established regarding its quantitative definition, including which tests to conduct and which cutoff criteria to apply. Therefore, further research is required to establish a quantitative definition for children with DLD across languages and cultures and enable comparisons of emotional competence in children with DLD from different linguistic and cultural backgrounds. In addition, emotional competence tele-interventions effectively improve the emotional competence of children with DLD. They are also feasible and acceptable for both children and their parents. These findings indicate that tele-interventions can be a viable option for individuals who lack access to in-person services due to hospital shutdowns or barriers related to transportation, location, or time. Visual materials such as images and videos of children’s interactions, implicit thoughts, and emotions should be used to understand and discuss emotional situations, with a preference toward realistic content. In addition, the language used in SSTIs should match the language capabilities of children. Although the results of our tele-intervention approach are satisfactory, future studies should explore its effectiveness in hospital settings to determine its real-world impact and examine broader mental health outcomes of tele-interventions for children with DLD. Further research is also required to address unresolved concerns regarding assessment, diagnosis, telephone consultation, internet support systems, and inconsistent intervention outcomes. Addressing these concerns can facilitate the expansion of tele-interventions to enhance other competencies in children with DLD and include a broader population with different medical conditions. Overall, this emotional competence tele-intervention, which can be used on pediatric populations, should be conducted as a participatory process involving children and their parents, with technology used to increase the convenience of the intervention, provided that efficacy is achieved. The findings show that using remote technology in home-based tele-interventions is effective, feasible, and well accepted. These interventions improve emotional skills in children with DLD and help support their parents.

## Appendix

Multimedia Appendix 1 Example of a social story video.

## Abbreviations

ANCOVA: analysis of covariance
ASD: autism spectrum disorder
BET: basic emotional term
CET: complex emotional term
CLDS-R: Child Language Disorder Scale–Revised
DLD: developmental language disorder
DSM-5: Diagnostic and Statistical Manual of Mental Disorders, Fifth Edition
ELT: Emotional Lexicon Test
ICC: intraclass correlation coefficient
NVI: Nonverbal Index
SSTI: social story tele-intervention
TLD: typical language development
VAI: Vocabulary Acquisition Index
VCI: Verbal Comprehension Index

## References

[1] American Psychiatric Publication (2013). Diagnostic and Statistical Manual of Mental Disorders, Fifth Edition.

[2] Bishop DV, Snowling MJ, Thompson PA, Greenhalgh T, The CATALISE-2 Consortium (2017). Phase 2 of CATALISE: a multinational and multidisciplinary Delphi consensus study of problems with language development: Terminology. J Child Psychol Psychiatry.

[3] Bishop DV, Snowling MJ, Thompson PA, Greenhalgh T, CATALISE Consortium (2016). CATALISE: a multinational and multidisciplinary Delphi consensus study. Identifying language impairments in children. PLoS One.

[4] Samson AC, van den Bedem NP, Dukes D, Rieffe C (2020). Positive aspects of emotional competence in preventing internalizing symptoms in children with and without developmental language disorder: a longitudinal approach. J Autism Dev Disord.

[5] Spackman MP, Fujiki M, Brinton B (2006). Understanding emotions in context: the effects of language impairment on children's ability to infer emotional reactions. Int J Lang Commun Disord.

[6] Bahn D, Vesker M, Schwarzer G, Kauschke C (2021). A multimodal comparison of emotion categorization abilities in children with developmental language disorder. J Speech Lang Hear Res.

[7] Aznar A, Tenenbaum HR (2013). Spanish parents' emotion talk and their children's understanding of emotion. Front Psychol.

[8] Brinton B, Fujiki M, Hurst NQ, Jones ER, Spackman MP (2015). The ability of children with language impairment to dissemble emotions in hypothetical scenarios and natural situations. Lang Speech Hear Serv Sch.

[9] Rieffe C, Dirks E, van Vlerken W, Veiga G (2016). The empathic mind in children with communication impairments: the case of children who are deaf or hard of hearing (DHH); children with an autism spectrum disorder (ASD); and children with specic language impairments (SLI). Theory of Mind Development in Context.

[10] Beck L, Kumschick IR, Eid M, Klann-Delius G (2012). Relationship between language competence and emotional competence in middle childhood. Emotion.

[11] Grau-Husarikova E, Sánchez Pedroche A, Mumbardó-Adam C, Sanz-Torrent M (2024). How language affects social cognition and emotional competence in typical and atypical development: a systematic review. Int J Lang Commun Disord.

[12] Yuill N, Little S (2018). Thinking or feeling? An exploratory study of maternal scaffolding, child mental state talk, and emotion understanding in language-impaired and typically developing school-aged children. Br J Educ Psychol.

[13] Salmon K, O'Kearney R, Reese E, Fortune CA (2016). The role of language skill in child psychopathology: implications for intervention in the early years. Clin Child Fam Psychol Rev.

[14] Kidwell SL, Young ME, Hinkle LD, Ratliff AD, Marcum ME, Martin CN (2010). Emotional competence and behavior problems: differences across Preschool Assessment of Attachment classifications. Clin Child Psychol Psychiatry.

[15] van den Bedem NP, Dockrell JE, van Alphen PM, Kalicharan SV, Rieffe C (2018). Victimization, bullying, and emotional competence: longitudinal associations in (pre)adolescents with and without developmental language disorder. J Speech Lang Hear Res.

[16] Chen HJ, Ko MHJ, Li ST, Chiu NC, Hung KL (2020). Prevalence of preschool children developmental disabilities in northeastern Taiwan - screening with Taipei City Developmental Screening Checklist for Preschoolers, 2nd Version. J Formos Med Assoc.

[17] Wu S, Zhao J, de Villiers J, Liu XL, Rolfhus E, Sun X, Li X, Pan H, Wang H, Zhu Q, Dong Y, Zhang Y, Jiang F (2023). Prevalence, co-occurring difficulties, and risk factors of developmental language disorder: first evidence for Mandarin-speaking children in a population-based study. Lancet Reg Health West Pac.

[18] Sheng L, Yu J, Su P, Wang D, Lu TH, Shen L, Hao Y, Lam BP (2023). Developmental language disorder in Chinese children: a systematic review of research from 1997 to 2022. Brain Lang.

[19] Fong R, Tsai CF, Yiu OY (2021). The implementation of telepractice in speech language pathology in Hong Kong during the COVID-19 pandemic. Telemed J E Health.

[20] Lai DC, Tseng YC, Guo HR (2018). Characteristics of young children with developmental delays and their trends over 14 years in Taiwan: a population-based nationwide study. BMJ Open.

[21] Test DW, Richter S, Knight V, Spooner F (2010). A comprehensive review and meta-analysis of the social stories literature. Focus Autism Other Dev Disabl.

[22] Leaf JB, Oppenheim-Leaf ML, Leaf RB, Taubman M, McEachin J, Parker T, Waks AB, Mountjoy T (2015). What is the proof? A methodological review of studies that have utilized social stories. Educ Train Autism Dev Disabil.

[23] Wahman CL, Pustejovsky JE, Ostrosky MM, Santos RM (2019). Examining the effects of social stories™ on challenging behavior and prosocial skills in young children: a systematic review and meta-analysis. Top Early Child Spec Educ.

[24] O’Connor KM, Hayes B (2019). A real-world application of social stories as an intervention for children with communication and behaviour difficulties. Emot Behav Diffic.

[25] Dessai RD (2012). Effectiveness of social stories in children with semantic pragmatic disorder. Adv Life Sci Technol.

[26] Schneider N, Goldstein H (2009). Social stories™ improve the on-task behavior of children with language impairment. J Early Interv.

[27] Bernad-Ripoll S (2007). Using a self-as-model video combined with social stories™ to help a child with Asperger syndrome understand emotions. Focus Autism Other Dev Disabl.

[28] Hoemann K, Xu F, Barrett LF (2019). Emotion words, emotion concepts, and emotional development in children: a constructionist hypothesis. Dev Psychol.

[29] Zimmerman KN, Ledford JR, Gagnon KL, Martin JL (2019). Social stories and visual supports interventions for students at risk for emotional and behavioral disorders. Behav Disord.

[30] Wellman HM, Peterson CC (2013). Deafness, thought bubbles, and theory-of-mind development. Dev Psychol.

[31] Paynter J, Peterson CC (2013). Further evidence of benefits of thought-bubble training for theory of mind development in children with autism spectrum disorders. Res Autism Spectr Disord.

[32] Blair C, Diamond A (2008). Biological processes in prevention and intervention: the promotion of self-regulation as a means of preventing school failure. Dev Psychopathol.

[33] Raver C (2002). Emotions matter: making the case for the role of young children's emotional development for early school readiness. Soc Policy Report.

[34] Castilla-Earls A, Bedore L, Rojas R, Fabiano-Smith L, Pruitt-Lord S, Restrepo MA, Peña E (2020). Beyond scores: using converging evidence to determine speech and language services eligibility for dual language learners. Am J Speech Lang Pathol.

[35] Chen CI, Chen RH (2013). Wechsler Preschool and Primary Intelligence Acale -Fourth Edition (Mandarin-Chinese version): Technical Manual.

[36] Lin BG, Huang YC, Huang GC, Hsuan CH (2008). Child Language Disorder Scale-Revised (Preschool Version): Administration Manual.

[37] Grazzani I, Ornaghi V (2012). How do use and comprehension of mental-state language relate to theory of mind in middle childhood?. Cogn Dev.

[38] Sturrock A, Freed J (2022). Preliminary data on the development of emotion vocabulary in typically developing children (5-13 years) using an experimental psycholinguistic measure. Front Psychol.

[39] Grazzani I, Ornaghi V, Riva Crugnola C (2015). Emotion comprehension and attachment: a conversational intervention with school-aged children. Eur Rev Appl Psychol.

[40] Grazzani IG, Ornaghi V, Piralli F (2011). Theory of mind and children's comprehension of psychological lexicon: preliminary data from the validation of Test of Emotional Lexicon (TLE). Psicologia Clinica Dello Sviluppo.

[41] Cohen J (1969). Statistical Power Analysis for the Behavioral Sciences.

[42] Verma JP, Verma P (2020). Determining sample size in general linear models. Determining Sample Size and Power in Research Studies.

[43] Letourneau NL, Duffett-Leger L, Levac L, Watson B, Young-Morris C (2011). Socioeconomic status and child development: a meta-analysis. J Emot Behav Disord.

[44] Streubel B, Gunzenhauser C, Grosse G, Saalbach H (2020). Emotion-specific vocabulary and its contribution to emotion understanding in 4- to 9-year-old children. J Exp Child Psychol.

[45] Causadias JM (2013). A roadmap for the integration of culture into developmental psychopathology. Dev Psychopathol.

[46] Bishop DV (2017). Why is it so hard to reach agreement on terminology? The case of developmental language disorder (DLD). Int J Lang Commun Disord.

[47] Ziegenfusz S, Paynter J, Flückiger B, Westerveld MF (2022). A systematic review of the academic achievement of primary and secondary school-aged students with developmental language disorder. Autism Dev Lang Impair.

[48] Black MM, Walker SP, Fernald LC, Andersen CT, DiGirolamo AM, Lu C, McCoy DC, Fink G, Shawar YR, Shiffman J, Devercelli AE, Wodon QT, Vargas-Barón E, Grantham-McGregor S (2017). Early childhood development coming of age: science through the life course. The Lancet.

[49] Britto PR, Lye SJ, Proulx K, Yousafzai AK, Matthews SG, Vaivada T, Perez-Escamilla R, Rao N, Ip P, Fernald LC, MacMillan H, Hanson M, Wachs TD, Yao H, Yoshikawa H, Cerezo A, Leckman JF, Bhutta ZA (2017). Nurturing care: promoting early childhood development. The Lancet.

[50] Guyer AE, Pérez-Edgar K, Crone EA (2018). Opportunities for neurodevelopmental plasticity from infancy through early adulthood. Child Dev.

[51] Causadias JM, Cicchetti D (2018). Cultural development and psychopathology. Dev Psychopathol.

[52] Ryou HW, Lee MJ, Kim JK, Choe MS (2013). Inadequate post-hoc statistical power analysis in Journal of the Korean Society of Emergency Medicine. J Korean Soc Emerg Med.

[53] Zvoch K (2016). The use of piecewise growth models to estimate learning trajectories and RtI instructional effects in a comparative interrupted time-series design. Elementary School J.

[54] Adrian JE, Clemente RA, Villanueva L, Rieffe C (2005). Parent-child picture-book reading, mothers' mental state language and children's theory of mind. J Child Lang.

[55] Frizelle P, McKean C, Eadie P, Ebbels S, Fricke S, Justice LM, Kunnari S, Leitão S, Morgan AT, Munro N, Murphy CA, Storkel HL, Van Horne AO (2023). Editorial perspective: maximising the benefits of intervention research for children and young people with developmental language disorder (DLD) - a call for international consensus on standards of reporting in intervention studies for children with and at risk for DLD. J Child Psychol Psychiatry.

[56] Kavussanu M, Barkoukis V, Hurst P, Yukhymenko-Lescroart M, Skoufa L, Chirico A, Lucidi F, Ring C (2022). A psychological intervention reduces doping likelihood in British and Greek athletes: a cluster randomized controlled trial. Psychol Sport Exerc.

[57] Blanton M, Stroud R, Stephens A, Gardiner AM, Stylianou DA, Knuth E, Isler-Baykal I, Strachota S (2019). Does early algebra matter? The effectiveness of an early algebra intervention in grades 3 to 5. Am Educ Res J.

[58] Raudenbush S, Bryk A, Cheong Y, Congdon R, du Toit M (2011). HLM 7: Hierarchical Linear and Nonlinear Modeling.

[59] Rhoads C (2016). Coherent power analysis in multilevel studies using parameters from surveys. J Educ Behav Stat.

[60] Chen CC, Barnhart HX (2008). Comparison of ICC and CCC for assessing agreement for data without and with replications. Comput Stat Data Anal.

[61] Ise E, Schröder S, Breuer D, Döpfner M (2015). Parent-child inpatient treatment for children with behavioural and emotional disorders: a multilevel analysis of within-subjects effects. BMC Psychiatry.

[62] Aarts S, van den Akker M, Winkens B (2014). The importance of effect sizes. Eur J Gen Pract.

[63] Zhang Z, Yuan KH (2018). Practical Statistical Power Analysis using WebPower and R.

[64] Grosse G, Streubel B, Gunzenhauser C, Saalbach H (2021). Let's talk about emotions: the development of children's emotion vocabulary from 4 to 11 years of age. Affect Sci.

[65] De Koning BB, van der Schoot M (2013). Becoming part of the story! Refueling the interest in visualization strategies for reading comprehension. Educ Psychol Rev.

[66] Brinton B, Fujiki M (2011). Emotion talk: helping caregivers facilitate emotion understanding and emotion regulation. Top Lang Disord.

[67] Burns MK, Codding RS, Boice CH, Lukito G (2019). Meta-analysis of acquisition and fluency math interventions with instructional and frustration level skills: evidence for a skill-by-treatment interaction. School Psychol Rev.

[68] Bell ML, Whitehead AL, Julious SA (2018). Guidance for using pilot studies to inform the design of intervention trials with continuous outcomes. Clin Epidemiol.

[69] Teresi JA, Yu X, Stewart AL, Hays RD (2022). Guidelines for designing and evaluating feasibility pilot studies. Med Care.

[70] Keys EM, Norris JM, Cameron EE, Bright KS, Tomfohr-Madsen LM, Benzies KM (2019). Recruitment and retention of fathers with young children in early childhood health intervention research: a systematic review and meta-analysis protocol. Syst Rev.

[71] Liverpool S, Mota CP, Sales CM, Čuš A, Carletto S, Hancheva C, Sousa S, Cerón SC, Moreno-Peral P, Pietrabissa G, Moltrecht B, Ulberg R, Ferreira N, Edbrooke-Childs J (2020). Engaging children and young people in digital mental health interventions: systematic review of modes of delivery, facilitators, and barriers. J Med Internet Res.

[72] Gartlehner G, Hansen RA, Nissman D, Lohr KN, Carey TS (2006). A simple and valid tool distinguished efficacy from effectiveness studies. J Clin Epidemiol.

[73] Chorpita BF, Daleiden EL, Ebesutani C, Young J, Becker KD, Nakamura BJ, Phillips L, Ward A, Lynch R, Trent L, Smith RL, Okamura K, Starace N (2011). Evidence‐based treatments for children and adolescents: an updated review of indicators of efficacy and effectiveness. Clin Psychol Sci Pract.

[74] Frizelle P, McGill M (2022). Prologue to the forum: speech and language tele-intervention: the future is now. Lang Speech Hear Serv Sch.

[75] Nelson LH, Rudge AM, Dawson P, Culianos D, Stredler-Brown A (2022). Provider perspectives in serving children who are deaf or hard of hearing and their families using tele-intervention. J Early Hear Detect Interv.

[76] Rudge AM, Brooks BM, Stredler-Brown A (2022). Working with families of young children who are deaf or hard of hearing through tele-intervention. J Early Hear Detect Interv.

[77] Lentejas KG, Lam JH, Tong SX (2022). Professional training and therapeutic resources needed for the adoption of telepractice in the Philippines. Commun Disord Q.

[78] Lowman JJ, Kleinert HL (2017). Adoption of telepractice for speech-language services: a statewide perspective. Rural Special Educ Q.

[79] Fairweather GC, Lincoln MA, Ramsden R (2016). Speech-language pathology teletherapy in rural and remote educational settings: decreasing service inequities. Int J Speech Lang Pathol.

[80] Wei WJ (2019). Development and evaluation of an emotional lexicon system for young children. Microsyst Technol.

[81] Chan M, Williams AI, Teng YP, Zhou Q (2022). Links between parent-child emotion talk and preschoolers’ socioemotional behaviors in Chinese-heritage families. Early Educ Dev.

[82] Ogren M, Sandhofer CM (2021). Emotion words in early childhood: a language transcript analysis. Cogn Dev.

