# Supplementary figures and images for: Efficacy, Feasibility, and Acceptability of an Emotional Competence Tele-Intervention for Mandarin-Speaking Children Aged 5 to 7 Years With Developmental Language Disorder: Pilot Study With an Interrupted Time-Series Design
Source: JMIR Pediatr Parent. 2025 Feb 11;8:e60333. doi: 10.2196/60333 (PMC11862772; doi:10.2196/60333)

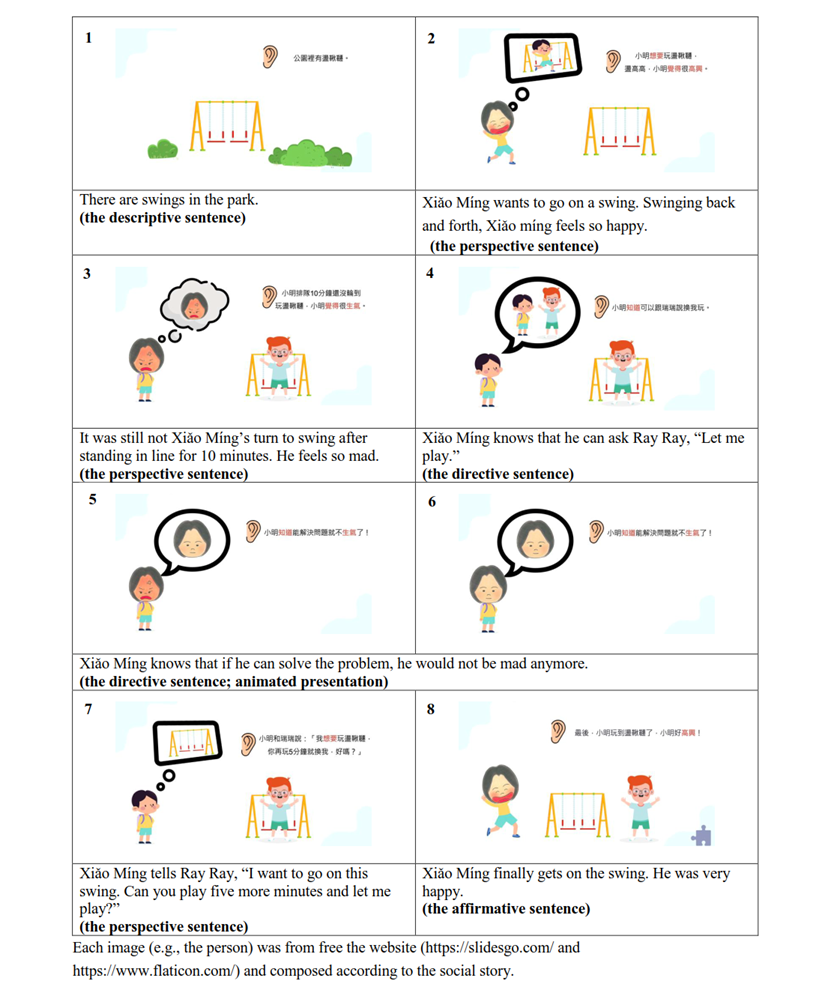

Supplement: Multimedia Appendix 1 [file pediatrics_v8i1e60333_app1.png]
